# Primary Peritoneal Low-Grade Serous Carcinoma in a 16-Year-Old Female: A Case Report

**DOI:** 10.3390/jcm15062343

**Published:** 2026-03-19

**Authors:** Yuang An, Yijian Fan, Yu Xia

**Affiliations:** Department of Ultrasound, State Key Laboratory of Complex Severe and Rare Diseases, Peking Union Medical College Hospital, Chinese Academy of Medical Sciences & Peking Union Medical College, Beijing 100730, China; anyuang@pumch.cn (Y.A.);

**Keywords:** primary peritoneal carcinoma, low-grade serous carcinoma, adolescents, rectal peritoneum, case report

## Abstract

**Background**: Primary peritoneal carcinoma (PPC) is an uncommon malignancy typically diagnosed in postmenopausal women, accounting for less than 1% of all gynecologic cancers. Its occurrence in adolescents is extremely rare. We present a case of a 16-year-old female with low-grade serous carcinoma (LGSC) arising from the anterior rectal peritoneum, highlighting diagnostic challenges and therapeutic considerations. **Case Presentation**: A 16-year-old girl presented with a 7-day history of lower abdominal pain. Ultrasound revealed an 8 cm mixed cystic–solid pelvic mass anterior to the rectum. Laboratory tests showed elevated CA-125 (106 U/mL). Exploratory laparotomy demonstrated an 8 cm solid mass attached to the anterior rectal wall, extending into the right mesorectum with peritoneal nodules at the bladder reflection. The uterus and adnexa appeared grossly normal. Frozen section analysis revealed adenocarcinoma with psammoma body formation. Histopathology and immunohistochemistry confirmed low-grade serous carcinoma: PAX8(+), WT1(+), CK7(+), ER(60%), PR(40%), CK20(–), and P53 wild-type. Peritoneal washings contained rare malignant cells. Postoperatively, the patient underwent total abdominal hysterectomy, bilateral salpingo-oophorectomy, and omentectomy. Final pathology confirmed low-grade serous carcinoma involving the anterior rectal wall, bilateral adnexal surfaces, and peritoneum. She completed six cycles of adjuvant chemotherapy (paclitaxel + carboplatin, TC regimen). No recurrence was observed during follow-up. **Conclusions:** This case underscores the importance of considering PPC in the differential diagnosis of pelvic masses in young females, even when the ovaries appear normal.

## 1. Introduction

Primary peritoneal carcinoma (PPC) is a rare epithelial malignancy that originates from the peritoneal lining and shares close histopathological, molecular, and clinical similarities with high- and low-grade serous ovarian carcinomas. Although PPC accounts for only a small proportion of all gynecologic malignancies, its incidence has increased slightly in recent years, largely due to improved recognition and classification criteria distinguishing it from primary ovarian cancer and peritoneal metastases [1,2].

The pathogenesis of PPC is closely linked to the secondary Müllerian system, with tumours thought to arise from multipotential mesothelial cells or from serous intraepithelial carcinomas of the fallopian tube that subsequently implant on the peritoneum [3]. Molecular profiling has revealed frequent KRAS and BRAF mutations in low-grade serous variants and TP53 alterations in high-grade lesions, mirroring the genetic patterns observed in ovarian serous carcinomas [4,5].

Clinically, PPC most commonly presents in postmenopausal women with symptoms such as abdominal distension, pain, or ascites. The disease tends to disseminate widely within the peritoneal cavity at presentation, mimicking advanced-stage ovarian carcinoma [6]. Despite its similarity to ovarian cancer, diagnostic criteria established by the Gynecologic Oncology Group (GOG) require minimal ovarian involvement and dominant extraovarian peritoneal disease. Differentiating PPC from primary ovarian carcinoma therefore remains a major diagnostic challenge, often requiring integrated clinical, imaging, and immunohistochemical evaluation using markers such as PAX8, WT-1, CK7, and CA-125.

PPC is exceedingly rare in adolescents and young women, with only sporadic cases documented in the literature [7]. Most reported cases occur in the fifth to seventh decades of life, and PPC localized to atypical sites, such as the anterior rectal peritoneum, has not been previously described. The current case therefore represents an exceptional presentation of low-grade serous PPC in a 16-year-old female, emphasizing the need for heightened clinical awareness of this rare entity even among young patients.

## 2. Case Presentation

A 16-year-old female, with no sexual history, presented to Peking Union Medical College Hospital (PUMCH) in August 2025 with a 7-day history of lower abdominal pain. She experienced menarche at the age of 12, with regular menstrual cycles of 15–30 days. The patient had undergone initial evaluation at a local hospital. Serum tumour marker analysis revealed an elevated CA125 level of 106 U/mL, while other tumour markers were within normal limits. Pelvic magnetic resonance imaging (MRI) demonstrated an 8 cm mixed cystic–solid mass in the pelvis, with irregular wall thickening, clear boundaries, and areas of cystic degeneration, necrosis, and hemorrhage. Transabdominal pelvic ultrasound showed a cystic–solid mass approximately 8 cm in diameter in right adnexal.

**Figure 1:**
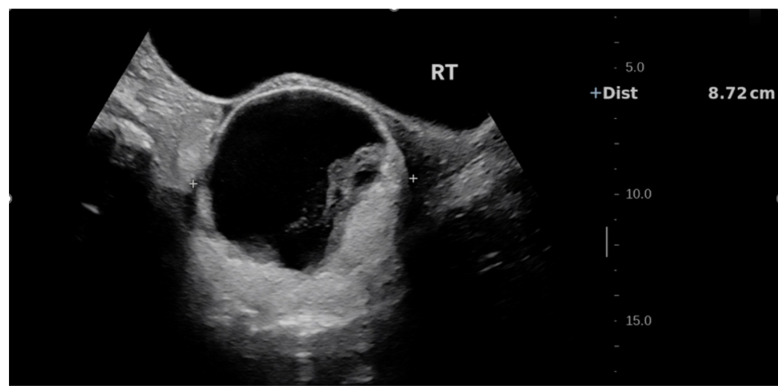
Transabdominal pelvic ultrasound revealed a right adnexal cystic–solid mass measuring 8.4 × 8.7 × 8.5 cm, demonstrating a mixed cystic–solid pelvic mass in the right adnexal region. The center of the lesion is predominantly cystic with anechoic areas. The lesion shows irregular internal walls with patchy hyperechoic areas. The right ovary is not clearly visualized.

**Figure 2:**
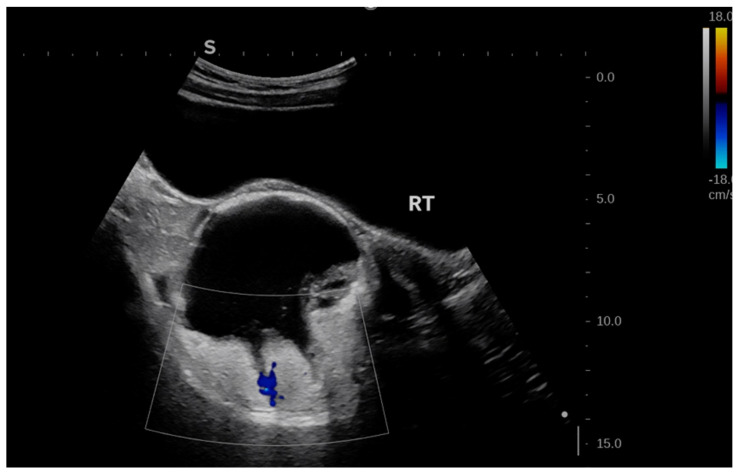
Colour Doppler flow imaging shows the vascular signals within the hyperechoic areas. No distinct blood flow signals were observed within the anechoic areas.

On physical examination at PUMCH, a firm, poorly mobile pelvic mass measuring approximately 8 cm in diameter was palpated posterior and to the right of the uterus, without tenderness. Repeat pelvic transabdominal ultrasound revealed a right adnexal cystic–solid mass measuring 8.4 × 8.7 × 8.5 cm, with clear margins but irregular inner walls and patchy hyperechoic areas within the cavity (Figure 1). Colour Doppler flow imaging detected mild strip-like vascular signals within the hyperechoic areas (Figure 2). The right ovary was not clearly visualized, while the left ovary and uterus appeared normal.

The patient subsequently underwent exploratory laparotomy and pelvic tumour resection in late August 2025. Intraoperatively, a solid mass approximately 8 cm in diameter was identified anterior to the rectum, largely retroperitoneal, with its surface covered by small tumour nodules and fixed adhesions to the pelvic floor. The uterus and bilateral adnexa appeared grossly normal, while small nodules were noted on the bladder peritoneal reflection. Frozen section analysis revealed fibrous tissue infiltrated by adenocarcinoma with abundant psammoma bodies.

Postoperative paraffin section pathology confirmed low-grade serous carcinoma (LGSC), characterized by fibrous tissue infiltration with papillary and micropapillary structures and extensive psammoma body formation. Immunohistochemistry (IHC) showed: PAX8(+), CA-125(+), CK7(+), ER (strong, 60%), PR (strong, 40%), Ki-67 (30%), P16 (patchy+), CK20(–), P53 (wild-type), WT-1(+), and CDX2(–). Molecular testing was performed as part of the diagnostic workup. Both KRAS and BRAF mutations were tested and no mutations were detected. Peritoneal washing cytology revealed the presence of malignant cells. Pathology from the bladder peritoneum, greater omentum, and right pelvic wall nodules also demonstrated fibro-fatty tissue infiltrated by low-grade serous carcinoma.

After multidisciplinary discussion and thorough counseling with the patient and her parents, a total hysterectomy with bilateral salpingo-oophorectomy and omentectomy was performed in September 2025. The final pathology confirmed low-grade serous carcinoma involving the serosal surfaces of both ovaries and fallopian tubes, with preservation of the internal parenchyma. The uterus showed proliferative endometrium and chronic cervicitis. The Gynecologic Oncology Group (GOG) has developed criteria to define PPC: (1) Both ovaries are normal in size or enlarged by a benign process; (2) The involvement in extraovarian sites is greater than the involvement on the surface of either ovary; (3) Microscopically, the ovaries are not involved with the tumor or exhibit only serosal or cortical invasions with dimensions smaller than 5 × 5 mm; (4) The histopathological and cytological characteristics of the tumor are predominantly of the serous type [8,9]. The final pathology confirmed low-grade serous carcinoma involving the serosal surfaces of both ovaries and fallopian tubes, with no evidence of involvement in the internal ovarian parenchyma. Specifically, the tumor was confined to the ovarian serosa, and the internal ovarian tissue remained unaffected. This finding aligns with the diagnostic criteria for PPC.

The follow-up period was four months, during which the patient underwent six cycles of the TC regimen (paclitaxel 270 mg and carboplatin 450 mg). The TC regimen is a standard chemotherapy protocol commonly used in the treatment of low-grade serous carcinoma. The patient tolerated the treatment well, and close monitoring during follow-up showed no immediate signs of recurrence. However, due to the rarity of the disease and the short follow-up duration, long-term surveillance is recommended to assess the outcome and potential recurrence of the disease. The patient is still undergoing follow-up treatment. As of the most recent visit, the patient remains in good condition under continued surveillance.

## 3. Discussion

PPC is a rare malignancy that arises from the peritoneal mesothelium and is typically diagnosed at an advanced stage, owing to its nonspecific clinical presentation and extensive intra-abdominal dissemination at the time of detection. PPC primarily affects postmenopausal women, although a few isolated cases have been described in adolescents and young adults. Histologically, PPC is almost indistinguishable from high- or low-grade serous ovarian carcinoma, but it develops in the absence of significant ovarian parenchymal involvement [6].

The exact pathogenesis of PPC remains uncertain, but mounting evidence supports two main theories: (1) the Müllerian metaplasia hypothesis, which proposes that pluripotent mesothelial cells undergo Müllerian transformation into serous epithelium capable of malignant transformation; and (2) the tubal origin hypothesis, suggesting that serous tubal intraepithelial carcinoma (STIC) cells from the fimbrial end of the fallopian tube exfoliate and implant on the peritoneal surface [1,3]. Recent molecular analyses have shown that low-grade serous variants of PPC often harbor KRAS or BRAF mutations, whereas high-grade variants are frequently associated with TP53 mutations, paralleling the genetic landscape of their ovarian counterparts [5].

This case represents PPC in an adolescent female arising from the anterior rectal peritoneum. The rare localization of the tumour in this case presented significant diagnostic challenges. Initially, the mass was misinterpreted as a possible ovarian or mesenteric lesion, given its position in the right adnexal region. Imaging modalities, including ultrasound and MRI, played an important role in identifying the tumour’s extragonadal origin and distinguishing it from primary ovarian malignancies [6]. Imaging plays an essential role in the evaluation of peritoneal malignancies and is critical for both diagnosis and surgical planning. Typical radiologic findings associated with peritoneal involvement include ascites, peritoneal nodules or thickening, omental infiltration (“omental cake”), and mesenteric implants on CT and MRI, which reflect the seeding pattern of malignant cells along peritoneal surfaces. These features are common to both PPC and peritoneal carcinomatosis from other primary sites (e.g., ovarian, gastrointestinal), making radiologic differentiation challenging without correlation with pathology [10].

In this case, the imaging pattern predominantly demonstrated peritoneal involvement with nodular implants and serosal thickening, without clear evidence of a dominant ovarian mass, supporting a primary peritoneal origin. Although imaging alone cannot definitively distinguish PPC from secondary peritoneal spread, the absence of a large adnexal primary lesion on CT/MRI combined with significant peritoneal disease raises suspicion for PPC and guided subsequent surgical and pathological evaluation [11]. Correlation with final pathology, which confirmed serosal involvement of the ovaries with preserved ovarian parenchyma and extensive peritoneal disease, further substantiates the primary peritoneal diagnosis. This imaging–pathology concordance reinforces the interpretation that the tumor originates from the peritoneum rather than representing peritoneal spread from an occult ovarian primary.

Additionally, advanced imaging techniques such as diffusion-weighted MRI and PET/CT have been reported to improve detection and characterization of peritoneal lesions and may be useful adjuncts in selected cases to better delineate disease extent preoperatively [11].

Given the patient’s young age (16 years), fertility preservation and long-term endocrine consequences were critical considerations in treatment planning. Prior to definitive surgery, comprehensive counseling was conducted with the patient and her family, including detailed discussions regarding reproductive potential, possible fertility-sparing strategies, oncologic risks, and long-term hormonal implications. Fertility-sparing approaches, such as unilateral salpingo-oophorectomy with preservation of the uterus and contralateral adnexa, are occasionally considered in selected early-stage low-grade serous tumors. However, in the present case, intraoperative findings demonstrated extensive peritoneal involvement and bilateral adnexal surface disease, raising significant concern for residual disease if conservative surgery were pursued. Given the distribution and extent of disease, the risk of undertreatment and subsequent recurrence was considered substantial. Therefore, definitive surgical management with hysterectomy and bilateral salpingo-oophorectomy was recommended to achieve maximal cytoreduction and optimize oncologic safety. The potential consequences of premature surgical menopause, including infertility, endocrine insufficiency, bone health risks, and psychosocial impact, were thoroughly discussed. After multidisciplinary consultation and careful deliberation, the patient and her guardians jointly elected to proceed with radical surgery, prioritizing oncologic control over fertility preservation.

Although PPC shares many clinical and histopathologic features with epithelial ovarian cancer, it is much less common and typically occurs in older, postmenopausal women. Population-based data show that PPC incidence rises with age, and most diagnoses occur in women around the 60s, similar to the age distribution of ovarian epithelial malignancies, suggesting that age-related factors contribute to carcinogenesis in the peritoneum and Müllerian epithelium. This age predilection may reflect cumulative DNA damage, age-associated decline in immune surveillance, and lifetime exposure to reproductive and environmental influences that affect peritoneal surface cells. However, unlike classic hormone-driven cancers such as breast and endometrial carcinoma, the direct role of circulating estrogens in PPC development remains unclear. While prolonged estrogen exposure (for example through menopausal hormone therapy) has been associated with increased risk of some epithelial ovarian tumors, the overall etiologic role of estrogen in PPC is less well established, and PPC does not demonstrate a clear estrogen-dependent growth pattern as seen in other hormone-dependent malignancies. Epidemiologic studies suggest divergent risk factor associations between invasive serous PPC and epithelial ovarian cancer, implying potential differences in underlying molecular pathways beyond hormone effects [12].

Genetic predisposition also plays an important role: germline mutations in BRCA1 and BRCA2 are established risk factors not only for ovarian and fallopian tube cancers but also for primary peritoneal carcinoma. Women carrying pathogenic BRCA mutations have a substantially increased lifetime risk of developing peritoneal serous malignancies, similar to their elevated risk for ovarian cancer, underscoring the relevance of familial cancer syndromes in PPC pathogenesis [13].

This case highlights the ethical and clinical complexity of managing peritoneal or low-grade serous malignancies in adolescents, where balancing oncologic radicality and reproductive preservation requires individualized, multidisciplinary, and family-centered decision-making.

LGSC exhibits distinctive features, including papillary and micropapillary structures, low mitotic activity, and the presence of psammoma bodies, which are characteristic of serous differentiation. The immunohistochemical profile of this patient—PAX8(+), WT1(+), CK7(+), ER(+), and PR(+) with CK20(–) and wild-type P53—is consistent with Müllerian-type differentiation and typical of peritoneal serous carcinoma [14].

PPC in adolescents is extremely rare. A notable case reported by Shibata et al. describes a child diagnosed with extraovarian primary peritoneal carcinoma [15]. This case provides one of the very few documented instances of PPC in a pediatric patient, underscoring its rarity in this demographic. In comparison, our case represents a unique manifestation of PPC with a rectal peritoneal origin, a feature not previously documented in pediatric PPC cases. While Shibata’s case highlights the involvement of the peritoneum without significant ovarian contribution, our case presents with bilateral adnexal serosal involvement. Despite the differences in tumor origin, both cases emphasize the challenges in diagnosing PPC in young patients, where clinical features may overlap with other abdominal malignancies, complicating accurate classification. Standard management includes cytoreductive surgery followed by platinum-based chemotherapy—most commonly paclitaxel combined with carboplatin (TC regimen)—as in this case [16].

Although the follow-up period in this case was six months, this duration is relatively short for an indolent malignancy such as PPC, and it does not allow for a comprehensive assessment of long-term prognosis and recurrence risk. PPC typically has a prolonged survival time, but recurrences may occur years after the initial treatment, which makes long-term follow-up essential. We acknowledge this limitation of the short follow-up duration, and future extended follow-up is necessary to better assess the long-term prognosis and potential recurrence of the disease.

This case underscores the importance of considering primary peritoneal carcinoma in the differential diagnosis of pelvic masses in young females, even when the ovaries appear morphologically normal. Comprehensive imaging and immunohistochemical assessment are indispensable for accurate diagnosis and appropriate management.

## 4. Conclusions

This case represents an adolescent patient with primary peritoneal low-grade serous carcinoma involving the anterior rectal peritoneum. It highlights the need to consider PPC in the differential diagnosis of pelvic masses in young females. Early diagnosis, complete surgical excision, and platinum-based chemotherapy remain essential for improved outcomes.

## Data Availability

The data presented in this study are available on request from the corresponding author.
